# Breast cancer screening practices of safety net clinics: Results of a needs assessment study

**DOI:** 10.1186/1472-6874-11-9

**Published:** 2011-04-02

**Authors:** Richard C Palmer, Raquel Samson, Anamica Batra, Maria Triantis, Irene D Mullan

**Affiliations:** 1 Robert Stempel College of Public Health and Social Work. 11200 SW 8th Street, Miami, FL 33199, USA; 2 8757 Georgia Avenue, 10th Floor, Silver Spring, Maryland 20910, USA; 3 Cancer and Tobacco Initiatives, Montgomery County Department of Health and Human Services. 1335 Piccard Drive, Rockville, MD 20850, USA

## Abstract

**Background:**

For low income and uninsured populations, safety net clinics are an important source of health care, including preventive services such as mammography screening. However, little is known about how well breast health is coordinated within the safety net clinic environment and what barriers patients encounter.

**Methods:**

A needs assessment was conducted among eight community-based safety net clinics located in Montgomery County, Maryland to learn about breast cancer referral and screening procedures. Structured in-depth interviews were conducted with clinic staff during the summer of 2008.

**Results:**

Safety net clinics reported that they routinely identified women who need mammography screening and referred women to mammography screening facilities. However, clinics were not aware of the limited number of free or low cost mammography screening slots available in the county or the waiting time to receive mammography services. Overall, screening barriers were common in the safety net system and only a few procedures were in place to help women overcome these barriers.

**Conclusion:**

Safety net clinics face multiple barriers in providing and coordinating breast cancer screening services for low income or uninsured patients. These barriers prevent the efficient allocation of mammography screening services and prevent underserved women from accessing an important preventive health service.

## Background

Underserved women, which include both racial/ethnic minorities and the poor, have a greater chance of dying from breast cancer [1]. Widespread use of breast cancer screening has been found to be effective in reducing deaths from these cancers [2,3], yet underserved women are more likely to die from this disease [4,5]. These women are less likely to be screened [3,6], which results in later stage diagnosis and decreased survival rates [2,3,7].

Barriers identified that contribute to low screening rates for breast cancer among underserved women suggest that there are both personal and health care factors that influence participation in screening [8]. Personal barriers include lack of awareness or knowledge about cancer screening, embarrassment in participating in actual screening procedures, low trust in prevention, and fear of cancer [9,10]. Additional personal barriers that prevent underserved women from participating in screening include procrastination, social and cultural beliefs, and perceptions of discrimination in the health care system [9,11,12]. Barriers to mammography screening include lack of access to health care, having no insurance and/or out of pocket costs [12,13]. Further, observational studies have documented that failure to receive a provider recommendation is perhaps the strongest correlate of failure to participate in mammography screening [9,11,13-15].

For low income, uninsured and underserved minority populations, safety net clinics are an important source of health care and preventive services [1]. Safety nets are defined as a network of private or public providers that provide health care to individuals who otherwise cannot pay for health care services [16]. Safety net clinics are often considered the last resource for receiving health care and typically do not receive federal health care dollars as do federally qualified health centers. However, the term safety net clinic and community health center are often used interchangeably. At present there are about 44 million uninsured people in the United States who lack health care coverage and and use safety nets clinics or federally qualified health centers as their primary source of care [16,17].

Given the fiscal constraints in delivering preventive care and the barriers associated with finding access for individuals without insurance, clinics serving the uninsured and poor are often given the role of coordinating patient care [16-20]. Several observational studies show that the present safety net clinic system is generally fragmented and results in less optimal care for patients [19-21]. Although there has been considerable research conducted in community health centers, little research has been conducted within safety net clinics.

The purpose of the study was to conduct needs assessment aimed at identifying how safety net clinics located in Montgomery County, Maryland coordinate care regarding breast health and mammography. Data obtained by medical record abstraction from these safety net clinics in 2006 revealed that mammography screening rates were abysmally low (12%). Therefore, the goal of this needs assessment was to assess and identify challenges faced by the safety net clinics in providing breast health services for their patients and to identify recommendations that could be implemented as part of a community planning grant to increase access to mammography services in Montgomery County.

## Methods

### Setting

This study was conducted at eight safety net clinics (Montgomery Cares) in Montgomery County, Maryland during the summer of 2008 (July - September) that are in partnership with the Primary Care Coalition (PCC) of Montgomery County. PCC is a private, non-profit, charitable organization working with public/private partners to provide high-quality, accessible, equitable, efficient, and outcome-driven health care services for low-income, uninsured residents of Montgomery County.

### Recruitment of study participants

An introductory call was made by the PCC study director to the medical director of each participating clinic informing the clinic about the needs assessment and the need for conducting interviews at their clinics. Medical directors were asked to identify administrative staff and health care providers to participate in in-depth interviews. In all, a total of 20 clinic staff representing medical directors (n = 6), healthcare providers (n = 6), and administrative staff (n = 8) were interviewed

### Procedures

Structured face-to-face and telephone in-depth interviews were conducted by a trained female interviewer experienced in conducting formative research. The interviewer used a moderator guide which consisted of questions that represented topics identified from the literature as barriers to screening and questions created by the PCC study team. Three interview guides (medical director, healthcare provider, and administrative personnel) were developed for data collection purposes. Although moderator guides were fairly similar, guides were tailored based on input from clinical directors so that more time could be spent discussing appropriate topics with clinic staff members best able to provide insight on topics (i.e., administrative staff were not asked clinical practice questions; see Table 1). Interviews lasted approximately one hour and were audiotaped to insure accurate and complete capturing of responses and then transcribed verbatim. The interviewer also recorded participant responses and other observations in field notes.

**Table 1:** Moderator Guide and Question Domains

Question Domain	Admissions Clerks/Administrative Personnel	Medical Director/Clinic Director	Primary Care Providers
Administrative Processes	X	X	NA
Education/Counseling	X	X	X
Breast Cancer Screening Policies	NA	X	X
Screening Practices and Beliefs	NA	X	X
Diagnosis and Treatment	NA	X	X
Cultural Awareness/and Linguistics	X	X	X
Referral Protocols and Scheduling	X	NA	NA

### Analysis

The audiotapes, transcripts, and interviewer notes were reviewed by the interviewer for the data reduction and analysis process. First, the notes recorded by the interviewer were compared with the transcripts to insure the accuracy and completeness of the responses. Interviewer notes were used to clarify unclear responses and fill in missing data from the transcripts. If responses were still unclear, the interviewer contacted study participants by telephone to clarify responses. Next, data from every interviewee within each clinic was examined to identify convergence and divergence. If there was any inconsistency, the interviewer then contacted participants to clarify their statements. Next, responses were compared between clinics to identify common breast health practices and procedures and to identify differences.

## Results

This needs assessment was conducted with eight safety net community clinics serving primarily low income, uninsured, ethnically diverse residents in Montgomery County, Maryland. Between July 2007 and June 30, 2008, the clinics served 13,300 residents. Sixty-four percent of these patients were female, and thirty four percent were male. All of the patients were adults, 31% were between 19-39 years of age, and 59% were between 40 -64 years of age. Ethnically, half of the patients served were Hispanic/Latino and half were non-Hispanic. By racial identification 13% were Asian, 21% African American or of African descent, 23% white, 35% were classified as other and 8% were listed with race/ethnicity unknown.

**Figure 1:**
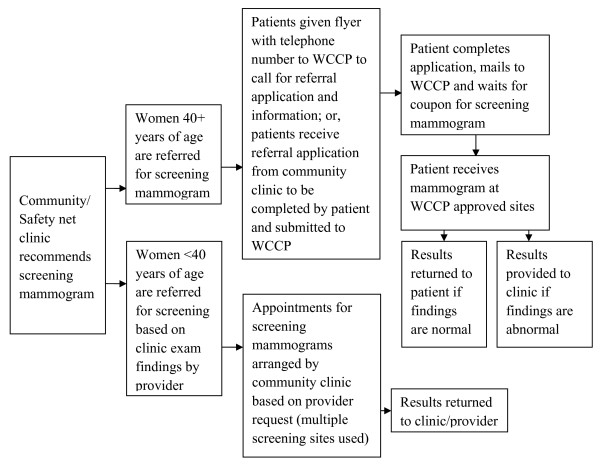
**Referral logistics for screening mammograms**.

The series of interviews conducted with clinic administrative and medical staff identified a consistent process of mammography referral across all eight clinics. Figure 1 documents the steps involved for women referred by a safety net clinic for mammography services in Montgomery County, MD. Predominately, clinics referred patients in need of mammography screening to the Women's Cancer Control Program (WCCP) for free mammograms. WCCP provides yearly breast and cervical cancer screening and follow-up for low income, uninsured/underinsured female residents of Montgomery County and is funded by the State of Maryland's National Breast and Cervical Cancer Early Detection Program [22].

During interviews, clinic staff discussed their perceptions of barriers to screening both at the clinic level and patient level during the interview process (Table 2). Overall, clinic staff perceived that cultural beliefs, cost, and fear of cancer were the main barriers for patients not receiving or participating in mammography screening. For mammography screening, clinics had the following procedures in place:

**Table 2:** Summary of findings explaining barriers to screening for breast cancer

**Perceived patient barriers**
Cultural beliefs (i.e., appropriateness of a male provider to provide breast care)
Screening costs
Religious beliefs
Fear of pain
Patient compliance and cooperation
Preference for a female health provider
Lack of familiarity with prevention as a health concept among many immigrants
Transportation
**Perceived clinical barriers**
Having a female provider at the clinic to conduct examinations
Access to care/cost
Location of mammogram facility
Availability of the mobile mammogram screening van at the clinic site
Time availability of providers for well-woman services at clinics
Screenings not conducted at the clinic
Inefficient WCCP paperwork requirements

### Referral Procedures

All clinics reported that they routinely identify women 40 and over who are in need of mammography screening. Clinics do not have onsite screening capabilities and therefore referred patients to WCCP. However, clinics did not assess if patients met eligibility requirements to qualify for WCCP services. Clinics were not aware of the limited number of WCCP mammography screening slots available, yet routinely referred patients to WCCP. Further, we found that clinics did not know how long it would take for a patient to obtain an appointment and receive screening at WCCP.

### WCCP Paperwork

For patients referred to WCCP, patients needed to complete eligibility paperwork. In general, clinics reported that patients found the WCCP paperwork to be complex, lengthy, difficult to read and understand, yet clinics did not provide any service to help patients with referral forms. Those who completed the paperwork and met eligibility criteria of WCCP often did not receive a mammogram due to limited WCCP screening slots.

### Screening Results

Clinics discussed that they had no way of documenting mammography results of women who were referred for mammography screening. Although clinics reported that they recommended and referred patients for screening routinely, there were no systems in place to track screening results. For women who had a WCCP sponsored mammogram, results were sent to the referring clinic only if results were abnormal. Commonly, clinics have to rely on the patient's self-report to document the screening results.

### Diagnosis and Treatment

Clinics reported a standard approach for diagnostic mammograms and treatment. All clinics reported that patients with abnormal changes in breast patterns between visits were given immediate appointments and referral services to appropriate medical care providers within Montgomery County. Diagnostic and treatment services are paid for by the State of Maryland's Breast and Cervical Cancer Diagnostic and Treatment Program. If patients were not eligible to participate in the State of Maryland's Breast and Cervical Cancer Diagnostic and Treatment Program, safety net clinics were expected to refer to a local community hospital or private physician's office that provides charitable care. However, once in treatment, clinics reported that there is no care coordination by them since patient cancer care is coordinated by the actual treatment site.

### Cultural and Linguistic Competence

The needs assessment identified cultural and linguistic issues that affected breast cancer screening. Clinic staff perceived cultural beliefs as barriers that negatively impact breast cancer screening among many clinic patients. Discussed unanimously across clinics were the ideas of fatalism and religiosity. Providers felt that many of their patients felt that they had no control of their health and that it was in "god's hands." Male providers also held the belief that Muslim female patients did not want them to discuss sensitive topics such as breast cancer screening with them, nor conduct examinations due to their religious beliefs. More than half of the clinics did not have formal cultural competency training for their staff. Only two clinics reported offering formal cultural competency training regularly. A language based medical translation service known as Language Link service was available to all the clinics and provided by PCC. Yet, clinics failed to make full use of Language Link service, resulting in improper patient-staff communication.

### Breast Health Education

All clinic staff reported that one-on-one education and written materials about breast cancer and the importance of mammography screening were available to patients. Further, clinics reported that providers did educate women about breast cancer and the important of screening during clinic visits. Several clinics reported having health educators available to patients. However, only half of the clinics reported having formal or established breast cancer education/counseling programs at their sites.

## Discussion

In conducting this needs assessment within the Montgomery Cares safety net system, we identified several issues that prevented racial and ethnically diverse uninsured and low-income women from receiving breast cancer screening. Further, we have identified possible solutions that could increase the efficiency and availability of breast cancer screening (Table 3) within Montgomery Cares system, and possibly, other safety net and community health centers in the United States. As more individuals become uninsured and use safety net clinics for their primary means of health care, demands on resources available require that safety net system become more efficient and better suited to coordinate patient care [21,23].

**Table 3:** Needs assessment recommendations

1. Development of a comprehensive referral tracking system that systematically documents the referral process from the clinic-level referral for screening through follow up to determine screening outcomes.
2. Development of a coherent application process that supports patient access to mammogram screening.
3. Development of guidelines to determine when all county clinics will make referrals for screening mammograms.
4. Ensure cultural and linguistic barriers to breast cancer prevention are addressed at time of mammogram referral.
5. Electronic Medical Record (CHL Care) implementation and training to support coordination of breast cancer screening care at the clinic level.
6. Ensure that the clinic environment is culturally and linguistically competent to provide effective, sensitive breast and cervical cancer health care prevention and treatment services for patients.
7. Evaluate the educational program and timing of the delivery of the breast health educational initiatives at each clinic and develop a protocol for best practices across clinics.

From our needs assessment findings, it is apparent that barriers to breast cancer screening exist within the Montgomery Cares safety net system. Perhaps the greatest barrier identified is the lack of care coordination between safety net clinics and the state-sponsored WCCP. This lack of care coordination resulted in long delays in screening and low screening rates. Only 12% of patients in Montgomery Cares who needed and were eligible to be screened actually were screened in 2006. Although alarming, the low percentage of women being routinely screened within the safety net system potentially reflects the acute care role that safety nets serve and not preventive health. However, the United States Preventive Services Task Force recommends that prevention counseling be part of every medical visit [24].

Overall, there is a lack of free or low cost breast cancer screening services available to low income and uninsured women nationally [13,22,25]. Similarly, WCCP in Montgomery County has limited capacity to screen the entire uninsured population that is in need of mammography screening services, yet Montgomery Cares clinics rely predominately on WCCP to provide screening since there are no alternatives available. Unfortunately this lack of capacity is a national problem and will require policy intervention to increase funding for low-income and uninsured women to receive life saving screening. WCCP has provided mammography services to approximately the same number of women from 2003-2007, yet the number of women needing screening has increased three-fold in the county.

Our needs assessment findings suggest that clinics across the United States that serve poor and uninsured patients should be aware of the length of time it takes for patients to receive mammography screening and should have alternative mammogram centers and resources available so that women can receive timely mammography screening. As a result of this needs assessment, PCC created a partnership with local hospital systems to develop a strategy that would provide low-cost/no cost mammograms. Further, PCC implemented a rapid referral process which has increased mammography screening among Montgomery Care patients. However, the demand still exceeds the resources available to ensure that all women needing a mammogram receive timely screening.

Further, we uncovered another reason why the rate of screening was so low among Montgomery Cares clinics. We found that patients were referred for mammography screening but were not systematically tracked by clinics. Often these women encountered barriers such as no availability at WCCP or high out-of-pocket costs which resulted in women not receiving and delaying screening. Additionally, this needs assessment identified that when a patient did receive screening, results were rarely sent to the clinics once the mammogram was completed.

Providers at all the safety net clinics noted that it was difficult to follow up with patients when results are not sent to the clinic after a patient is screened. Clinic directors and staff reported that they did not do anything to obtain the results of referred patients from mammogram providers due to time and workforce constraints. This sometimes led to women being referred for unnecessary screenings and duplicative services. Additionally, providers used self-report from patients to document completion of a mammogram on a subsequent visit to the clinic. Overall, procedures being used within the Montgomery Cares safety net system did not provide ongoing provision of follow-up care for those patients who may need additional imaging or diagnostic testing. Findings from the needs assessment suggest that the Montgomery Care safety net clinics need to develop a system to track and provide coordinated care to patients who have been referred for mammography.

The needs assessment also found that of the women who are eligible for screening through the WCCP, many do not have the ability to complete the application due to literacy barriers or unable to provide additional eligibility documentation to be accepted into the program. Clinics reported that they understood the WCCP eligibility paperwork was cumbersome, yet no effort was made to coordinate with WCCP to simplify the referral process and paperwork. Montgomery Cares clinics must develop a means to assist patients to complete this arduous and cumbersome paperwork and work with WCCP to streamline the paperwork needed.

The authors acknowledge the following limitations of the needs assessment. First, findings of this needs assessment may not be generalizable to other settings. Next, the needs assessment did not include the patient perspective and limits understanding of all of the personal barriers that may exist for patients within the safety net clinic system. Additionally, the study used a structured interview guide which may have limited exploration of ideas and findings. Nonetheless, even with these limitations, there are several strengths. First, there is a lack of literature of how safety net clinics coordinate care, especially breast cancer screening. More importantly, findings shed light on how system level factors play an integral role of preventing or delaying mammography screening in underserved populations in Montgomery County, and perhaps, other parts of the United States.

## Conclusion

This needs assessment suggests two areas in particular that need to be addressed: organizational change within safety net clinics (coordination of patient care) and policy level interventions to increase funding and access to mammograms (Table 3). Overall, our findings underscore the importance for increased access to mammography services for low income and uninsured populations and the need for standardized systems to provide comprehensive breast care in safety net systems. The lack of coordination between Montgomery Cares clinics and WCCP presented a significant barrier for care coordination and presented a unique burden to patients since they had to remember receiving a mammogram and their results. Further, given that all the safety clinics in Montgomery County referred to WCCP for mammograms for their patients, clinics need to better understand and monitor the referral process. A referral to a system that cannot provide care due to limited screening capacity has limited benefit to a patient and places considerable burden on a patient to identify a no cost screening site. Lastly, the arduous WCCP application processes proved to be a significant barrier for receiving mammography screening services. Efforts are needed to reduce and streamline paperwork to ensure that underserved populations are not systematically excluded from screening due cumbersome paperwork and inability to complete paperwork due to literacy barriers.

## Authors' contributions

RP conceptualized the study, participated in data collection, supervised data analysis, and drafted the manuscript. RS conceptualized the study, developed study materials, supervised data collection, and drafted the manuscript. AB performed data analysis and assisted with data interpretation. MT conceptualized the study and interpretation of data. IM conceptualized the study and interpretation of data. All authors helped review drafts of the manuscript and have read and approved the final manuscript.

## Author Disclosure Statement

None of the authors have any financial conflicts of interest to declare.

## Pre-publication history

The pre-publication history for this paper can be accessed here:

http://www.biomedcentral.com/1472-6874/11/9/prepub
